# A cross-sectional study of the prevalence and risk factors for hypertension in rural Nepali women

**DOI:** 10.1186/1471-2458-13-55

**Published:** 2013-01-21

**Authors:** Rumana J Khan, Christine P Stewart, Parul Christian, Kerry J Schulze, Lee Wu, Steven C LeClerq, Subarna K Khatry, Keith P West

**Affiliations:** 1 Program in International and Community Nutrition, University of California, Davis, CA, USA; 2 Program in Human Nutrition, Department of International Health Bloomberg, School of Public Health Johns Hopkins University, 615 North Wolfe Street, Baltimore, 21205, Maryland; 3 Nepal Nutrition Intervention Project-Sarlahi, Kathmandu, Nepal

**Keywords:** Blood pressure, Hypertension, Cardiovascular risk, Nepal, Rural

## Abstract

**Background:**

The prevalence of hypertension is increasing in much of the South Asian region, including Nepal. This paper reports the prevalence and risk factors of hypertension and pre-hypertension among adult women in a rural community of Nepal.

**Methods:**

Cross-sectional data on socioeconomic status (SES), lifestyle factors and blood pressure (BP) were collected from a cohort of 15,934 women in rural Nepal in 2006–08. Among a subsample (n = 1679), anthropometry and biomarkers of cardiovascular risk were measured.

**Results:**

The mean age of women was 34.2 years (range 16.4-71.2 years). More than three percent (3.3%) had hypertension and 14.4% had pre-hypertension. In an adjusted analysis, lower SES, especially lower household farm assets and storage of food for long term consumption, was associated with increased odds of hypertension (OR = 1.14 for mid-level SES and OR = 1.40 for low SES; p for trend < 0.01). Smoking, alcohol use and not working outside the home were also associated with higher risk. In a subsample, both systolic BP (SBP) and diastolic BP (DBP) were positively associated with high triglycerides (SBP β = 4.1 mm Hg; DBP β =3.6 mm Hg), high HbA1c (SBP β = 14.0; DBP β = 9.2), raised fasting glucose (SBP β = 10.0; DBP β = 6.9), high BMI (SBP β = 6.7; DBP β = 5.1) and high waist circumference (SBP β = 6.2; DBP β = 5.3) after adjusting for potential confounders (p for all <0.01).

**Conclusions:**

Although the prevalence of hypertension was low in this cohort, it was more prevalent among the poorer women and was strongly associated with other cardiovascular risks. These associations at a relatively young age may confer greater risk for cardiovascular disease among women in later life, indicating the need for interventions to reduce the progression from pre-hypertension to hypertension.

## Background

Low and middle-income countries bear a large burden of cardiovascular disease (CVD), accounting for 80% of the global CVD-related deaths and 87% of disability adjusted-life years lost [1]. CVD rapidly has become a major cause of mortality and morbidity in low income South Asian countries as well [2-4]. In developed countries, age adjusted death rates from CVD are declining due to preventive interventions and improved treatments [5]. Thus, CVD in developed countries is considered a disease of the aged, where only 23% of deaths occur below the age of 70 years; however, in South Asia, 52% of CVD deaths occur among people under 70 years [3,6]. Additionally, it has been estimated that the age of onset of acute myocardial infarction was an average of 6 years of age earlier in South Asia compared to other regions [7]. This trend has led to substantial loss of potential human productive years. Early identification and rigorous control of intermediate risk factors are needed to prevent and control CVD in this part of the world.

Hypertension is one of the leading risk factors for CVD and the prevalence of hypertension has been increasing in the South Asian region including Nepal [8]. Despite rapid urbanization, about 83% of Nepal’s inhabitants live in rural areas [9]. Few studies have attempted to describe the burden and determinants for hypertension in rural Nepal and such data are limited in the South Asian context. Exploration of such data in rural Nepal will help to understand the etiology of CVD in a population at the cusp of the epidemiologic and nutrition transition, with findings that may be generalizable to other parts of rural South Asia.

The primary aim of this paper is to explore the prevalence and risk factors for hypertension and pre-hypertension among adult women, previous participants of a nutrition intervention trial [10] in a rural community of Nepal. We also report the prevalence of other CVD risk factors, such as obesity, cholesterol, triglycerides, and HbA1c, and their association with blood pressure.

## Methods

This study utilizes cross-sectional data from a follow-up of women who had been enrolled in a double blind, placebo controlled, cluster randomized trial of vitamin A or β carotene supplementation provided to women for three years (1994–1997). Details of the trial were published previously [10]. Briefly, the study was conducted in the rural, low-lying Sarlahi District of Nepal. The study area comprises 30 Village Development Communities (VDCs), each divided into 9 administrative wards. Over a study period from July 1994 to June 1997, 17,531 infants were born to women enrolled in the trial. Women were supplemented before, during and after pregnancy throughout the study period. A subsample area was selected for more intensive monitoring during the trial. The area contained roughly 10% of the study population and selection was based on ease of access to clinics and a paved road in order to facilitate biochemical sample collection and to visit women at birth. The cohort of women who became pregnant during the trial period have been followed over time along with their children to monitor long-term health outcomes [11]. From 2006–2008, women were followed-up (n = 16,469) during a series of up to three household visits. The current analysis uses the data from this follow-up period (n = 15,934), excluding women who were currently pregnant (n = 470) or who did not know their pregnancy status (n = 65).

During the first visit, field workers conducted interviews to collect information on household socioeconomic status, literacy, ethnicity and occupation. Information on their smoking status and alcohol consumption were also collected. Blood pressure was measured four times on the right arm using an automated measurement device (BPM 300, BPTrue, Canada). The first measure was dropped and the mean of the last three was used for analysis. If the mean systolic or diastolic blood pressure (SBP and DBP, respectively) were ≥ 140/90, the measurements were repeated. Mid-upper arm circumference (MUAC) was measured on the upper left arm at the mid-point of the acromion process and the tip of the olecranon using a standard insertion tape.

During a second visit among a subsample of 1679 women, anthropometric measurements were recorded, including height (Harpenden stadiometer, Crosswell, UK), weight (Seca 881, Hamburg, Germany), and waist circumference (WC) (Seca 200, Hamburg, Germany). Women were then asked to fast overnight and were visited the following morning by a team of phlebotomists to collect venous blood. Total cholesterol (TC), HDL cholesterol, triglycerides (TG), and fasting glucose were measured in plasma specimens (LDX Analyzer, Cholestech, Hayward, CA). Of the women who consented to the blood draw (n = 1447), 251 (17.3%) had not fasted, defined as no food or drink other than water within 8 hours of the blood draw. Glucose data was only analyzed among women who were fasting (n = 1196).

Continuous variables such as age, SBP, DBP, MUAC, height, weight, BMI and WC were checked for normality, outliers and missing values. Blood pressure and lipid parameters were categorized using standard cutoffs [12,13]. Hypertension was present if SBP and/or DBP were ≥140/90 mm Hg [12]. Pre-hypertension was defined by SBP ≥120 mmHg but <140 mmHg and/or DBP ≥ 80 mmHg but <90 mmHg [12]. The cutoff for high TC was ≥5.17 mmol/L (200 mg/dL); high TG was ≥1.7 mmol/L (150 mg/dL); low HDL cholesterol was <1.03 mmol/L (40 mg/dL), raised fasting glucose was ≥5.6 mmol/L (100 mg/dL) and high HbA_1_c was ≥6.5% [14]. Overweight was defined as BMI ≥23 kg/m^2^ and abdominal obesity defined as WC ≥80 cm [15-17]. Women were also classified according to the International Diabetes Federation’s worldwide definition of the metabolic syndrome which includes a WC ≥80 cm plus ≥ 2 of the followings: 1) raised triglycerides: ≥1.7 mmol/L; 2) reduced HDL cholesterol: <1.03 mmol/L; 3) raised blood pressure: systolic BP ≥130 or diastolic BP ≥85 mm Hg; and 4) raised fasting plasma glucose: ≥5.6 mmol/L [16].

SES was analyzed using Principal Component Analysis (PCA) by examining 17 questions on SES, extracting three components with eigen values >1 which explained 41% of the variance. One component represented a summary measure of household quality and status, with the most heavily weighted variables including type of roof and walls, type of latrines, number of servants in the house, number of rooms in the house, having electricity, and owning a motorcycle. The second component represented household farming assets and food storage, with the most heavily weighted variables being kilograms of rice stored per household member, duration of time rice stores would last, ownership of cultivable land, and ownership of cattle, goats and bullock carts. The third component represented other household assets, with the most heavily weighted variables including bicycle, TV, radio and wristwatch ownership. Each of these three components of SES were categorized into tertiles based on their component scores.

The primary outcome measure for this analysis was blood pressure, evaluated both as a dichotomous and a continuous variable. The mean differences of BP among supplementation groups (vitamin A, β carotene or placebo), were within 0.5 mm Hg and the prevalence of hypertension and pre-hypertension did not differ between groups. Thus, all intervention groups were combined for this analysis. For the full sample, simple and multiple logistic regression analyses were done to study the relationship between each independent variable with the outcomes of hypertension or pre-hypertension. The independent variables included the three SES factors, age, smoking status (yes/no), alcohol consumption (yes/no), MUAC (in cm), ethnicity (women of Pahadi ethnicity historically originated from the hill areas of Nepal while those of Madheshi ethnicity originated from the plains) and occupation, which was grouped into two categories: 1) no reported work or study or 2) work or study outside home, including farmers, unskilled or contracted laborers, business, private or government service, and students. For both the regression models, effect modification by ethnicity and age were evaluated with likelihood ratio tests by comparing two nested multivariate models with and without the interaction term. The results were stratified if any significant interaction was found.

For the subsample, blood pressure was analyzed continuously, as the sample size was smaller and the number of women with hypertension was relatively low. In order to examine the associations of SBP and DBP with other cardiovascular risk factors (high TC, high TG, low HDL cholesterol, raised fasting glucose, high HbA_1_c, high BMI, high WC), simple linear regression was performed with SBP or DBP as the outcome variable and each of these risk factors as predictors. Those with normal values of the predictor variables were considered as a reference group. Multivariable regression models were adjusted for other predictors found to be significantly associated with hypertension in the full cohort. In addition, models examining the lipid profile, glucose and HbA_1_c were further adjusted for BMI.

The follow-up study was approved by the Institutional Review Boards at the Johns Hopkins Bloomberg School of Public Health and the Institute of Medicine in Kathmandu, Nepal.

## Results

The study sample included 15,934 women with a mean age of 34.2 years (Standard deviation or SD = 5.9), 60% of whom were between the age of 31–45 years. Roughly, one third of them were Pahadi and the rest were Madheshi. Most women could not read or write in any language (84.9%) and most worked outside the home (81.7%). Approximately 42.2% did not own any land; 49.7% had no electricity; and 14.5% owned no livestock. Around 22.5% of women were smokers and 11.2% were alcohol drinkers. Their mean MUAC was 23.1 cm (SD = 2.7).

The mean SBP was 104.3 mm Hg (SD = 12.3) and ranged from 68 to 216 mm Hg and the mean DBP was 70.9 mm Hg (SD = 9.3) and ranged from 48 to 122 mm Hg. A total of 530 (3.3%) women had hypertension while 2296 (14.4%) had pre-hypertension. Participant characteristics and selected SES variables by BP category are presented in Table 1. Notably, there was a trend of increasing prevalence of hypertension and pre-hypertension with increasing age and higher MUAC. Women with hypertension or pre-hypertension were more likely to be smokers and twice as likely to consume alcohol. Pahadi women had a greater probability of hypertension (4% versus 3%) or pre-hypertension (17% versus 13%) than Madheshi women. Women with hypertension or pre-hypertension were also more likely to come from households that did not own any land or livestock. Women working outside the home had a lower prevalence of both hypertension and pre-hypertension than women who stayed at home.

**Table 1:** Selected characteristics of adult Nepali women in the cohort in 2006–2008 by blood pressure categories

	**Missing values**	**Hypertension (n = 530)**	**Pre-hypertension (n = 2296)**	**Normal blood pressure (n = 13,108)**	**P-value**^**a**^
	n	n (%)			
**Age (years)**	11				<0.0001
16-30		109 (1.9)	657 (11.7)	4853 (86.4)	
31-45		366 (3.8)	1503 (15.6)	7742 (80.6)	
46 and above		55 (7.9)	134 (19.3)	504 (72.7)	
**Smoking**	11				<0.0001
No		353 (2.9)	1745 (14.1)	10246 (83.0)	
Yes		177 (4.9)	550 (15.4)	2852 (79.7)	
**Alcohol**	12				<0.0001
No		426 (3.0)	1922 (13.6)	11789 (83.4)	
Yes		104 (5.8)	372 (20.8)	1309 (73.3)	
**MUAC (cm)**^**b**^	0	23.7 (3.0)	23.7 (2.9)	23.0 (2.8)	<0.0001^c^
**Pahadi/Madheshi**	38				<0.0001
Pahadi		200 (3.9)	877 (17.0)	4068 (79.1)	
Madheshi		330 (3.1)	1410 (13.1)	9011 (83.8)	
**Occupation**	16				<0.0001
Does not work		126 (4.3)	468 (16.1)	2310 (79.5)	
Works outside the home		404 (3.1)	1826 (14.0)	10784 (82.9)	
**Literacy**	8				0.991
No		451 (3.4)	1949 (14.4)	11120 (82.2)	
Yes		79 (3.3)	347 (14.4)	1980 (82.3)	
**Land ownership**	63				<0.0001
No		254 (3.8)	1027 (15.3)	5414 (80.9)	
Yes		275 (3.0)	1253 (13.7)	7648 (83.3)	
**Livestock**	23				<0.0001
No		116 (5.1)	368 (16.1)	1799 (78.8)	
Yes		414(3.0)	1921 (14.1)	11293 (82.9)	
**TV**	31				0.104
No		364 (3.3)	1549 (14.0)	9143 (82.7)	
Yes		166 (3.4)	739 (15.2)	3942 (81.3)	
**Electricity**	23				0.357
No		270 (3.4)	1107 (14.0)	6529 (82.6)	
Yes		260 (3.2)	1181 (14.8)	6564 (82.0)	
**Composite indicators of SES**	577				
**Household quality**					
High		167 (3.2)	778 (15.1)	4218 (81.7)	0.332
Medium		178 (3.5)	695 (13.7)	4218 (82.9)	
Low		168 (3.3)	731 (14.3)	4204 (82.4)	
**Household Assets**					0.326
High		177 (3.4)	768 (14.8)	4227 (81.7)	
Middle		177 (3.5)	746 (14.6)	4198 (82.0)	
Low		159 (3.1)	690 (13.6)	4215 (83.2)	
**Household farming and food storage**^**d**^					
High		142 (2.8)	713 (13.8)	4305 (83.4)	<0.0001
Medium		166 (3.3)	685 (13.4)	4253 (83.3)	
Low		205 (4.0)	806 (15.6)	4082 (80.1)	

Abbreviations: SES = Socioeconomic Status; MUAC = Mid Upper Arm Circumference.

^a^Pearson Chi square except where noted.

^b^Mean (SD).

^c^One way ANOVA.

^d^p for chi square linear trend < 0.001 for hypertension and pre-hypertension.

Table 2 and Table 3 show the unadjusted and adjusted relationships of BP categories with socioeconomic and other predictors. In the adjusted analysis (Table 2) two measures of SES (household quality and household assets) were not associated with hypertension, but household farming assets and food storage was. Women with low status in this latter SES indicator had a significant 40% increased odds of hypertension compared to women of high status.

**Table 2:** Association between selected risk factors and prevalence of hypertension among adult Nepali women (n = 13,638)

	**Hypertension^a^**	
	**Odds Ratio (95% CI)**	
	**Unadjusted**	**Adjusted^b^**
**SES: Household quality**		
High	Reference	-
Middle	1.06 (0.86 to 1.32)	1.09 (0.87 to1.37)
Low	1.01 (0.81 to 1.26)	1.00 (0.78 to1.26)
**SES: Farming and food**		
High	Reference	-
Middle	1.18 (0.94 to 1.49)	1.14 (0.90 to 1.45)
Low	1.52 (1.22 to 1.89)	1.40 (1.12 to 1.76)
**SES: Household Assets**		
High	Reference	-
Middle	1.01 (0.81 to 1.24)	1.05 (0.84 to 1.31)
Low	0.90 (0.72 to 1.12)	0.92 (0.73 to 1.15)
**Age**		
16 to 30	Reference	-
31 to 45	2.10 (1.69 to 2.61)	2.00 (1.60 to 2.52)
46 and above	4.86 (3.47 to 6.80)	4.30 (3.09 to 6.26)
**Smoking**		
No	Reference	-
Yes	1.80 (1.50 to 2.17)	1.31 (1.10 to 1.58)
**Alcohol**		
No	Reference	-
Yes	2.20 (1.76 to 2.75)	1.53 (1.23 to 1.90)
**MUAC (cm)**	1.09 (1.06 to 1.13)	1.08 (1.05 to 1.12)
**Ethnicity**		
Pahadi	Reference	-
Madheshi	0.75 (0.62 to 0.89)	1.03 (0.83 to 1.27)
**Occupation**		
Does not work	Reference	-
Works outside home	0.69 (0.56 to 0.84)	0.65 (0.52 to 0.81)

Abbreviations: SES = Socioeconomic Status; MUAC = Mid Upper Arm Circumference.

^a^Hypertension defined as systolic blood pressure ≥ 140 mmHg and/ or diastolic blood pressure ≥ 90 mmHg.

^b^Adjusted for all other variables in the table.

**Table 3:** Association between selected risk factors and prevalence of pre-hypertension among adult Nepali women (n = 15,404)

	**Pre-Hypertension^a^**			
	**Odds Ratio (95% CI)**			
	**Pahadi**		**Madheshi**	
	**(n=4945)**		**(n=10,421)**	
	**Unadjusted**	**Adjusted^b^**	**Unadjusted**	**Adjusted^b^**
**SES: Household quality**				
High	Reference	-	-	-
Middle	1.08 (0.90 to 1.29)	1.04 (0.86 to 1.27)	0.84 (0.73 to 0.98)	0.92 (0.80 to 1.07)
Low	1.23 (1.03 to 1.46)	1.23 (1.03 to 1.52)	0.89 (0.75 to 1.01)	0.93 (0.79 to 1.08)
**SES: Farming and food**				
High	Reference	-	-	-
Middle	0.87 (0.71 to 1.03)	0.78 (0.69 to 1.12)	1.04 (0.90 to 1.20)	1.09 (0.95 to 1.27)
Low	1.03 (0.86 to 1.24)	0.95 (0.78 to 1.15)	1.28 (1.12 to 1.47)	1.31 (1.14 to 1.55)
**SES: Household Assets**				
High	Reference	-	-	-
Middle	1.11 (0.93 to 1.32)	1.11 (0.9 to 1.33)	0.94 (0.81 to 1.08)	0.99 (0.85 to 1.14)
Low	1.25 (1.04 to 1.51)	1.27 (1.04 to 1.55)	0.81 (0.67 to 0.96)	0.86 (0.74 to 1.00)
**Age**				
16 to 30	Reference	-	-	-
31 to 45	1.74 (1.48 to 2.05)	1.67 (1.40 to 1.98)	1.30 (1.14 to 1.47)	1.34 (1.17 to 1.52)
46 and above	1.58 (1.11 to 2.25)	1.63 (1.12 to 2.34)	2.23 (1.72 to 2.88)	2.47 (1.88 to 3.24)
**Smoking**				
No	Reference	-	-	-
Yes	1.15 (0.99 to 1.34)	0.96 (0.80 to 1.16)	1.05 (0.87 to 1.16)	0.93 (0.79 to 1.10)
**Alcohol**				
No	Reference	-	-	-
Yes	1.55 (1.33 to 1.81)	1.57 (1.30 to 1.88)	1.51 (1.07 to 2.12)	1.46 (1.02 to 2.10)
**MUAC (cm)**	1.07 (1.04 to 1.10)	1.11 (1.08 to 1.14)	1.08 (1.06 to 1.11)	1.08 (1.05 to 1.10)
**Occupation**				
Does not work	Reference	-	-	-
Works outside home	0.89 (0.72 to 1.10)	0.82 (0.65 to1.03)	0.77 (0.67 to 0.88)	0.80 (0.70 to 0.92)

Abbreviations: SES = Socioeconomic Status; MUAC = Mid Upper Arm Circumference.

^a^Pre-hypertension defined by systolic blood pressure ≥ 120 mmHg but ≤140 mmHg and/or diastolic blood pressure was ≥ 80 mmHg but ≤90 mmHg.

^b^Adjusted for all other variables in the table.

We found a significant interaction between ethnicity and socioeconomic status while assessing the association of pre-hypertension and its predictors. We therefore have stratified the findings for pre-hypertension by ethnicity (Table 3). In the case of pre-hypertension, Madheshi women with low farming assets and food storage had a significant 31% increased risk compared to women of high status (p for trend < 0.001). This association was not significant for Pahadi women. However, for Pahadi women those with low household quality and low household assets had increased odds of having pre-hypertension than those with high household quality (OR, 1.23 and 95% CI, 1.03-1.52) and high household assets (OR, 1.27 and 95% CI, 1.04-1.55). In addition to different indicators of SES, age, alcohol drinking, MUAC and occupation remained significantly associated both with hypertension and pre-hypertension in multivariable models. Smoking remained significant only for hypertension and not for pre-hypertension.

### Cardiovascular risk factors in the subsample

Among the subsample of 1679 women, there was a low prevalence of high TC and raised fasting glucose, yet a high prevalence of low HDL cholesterol (Table 4). About 10.2% women had high TG, about 12.0% were overweight and 14.0% of women had abdominal obesity. Only 5% of women had metabolic syndrome. The mean SBP and DBP were higher among women with high TC, high TG, high HbA_1_c, high BMI and high WC and raised fasting glucose compared to women with normal values (Table 4).

**Table 4:** Association between cardiovascular risk factors and mean blood pressure among adult Nepali women (N = 1679)

**Risk factors^a^**	**Prevalence**	**Systolic BP**		**Diastolic BP**	
	**N (%)**	**Beta^c^ (95% CI)**	**P value**	**Beta^c^ (95% CI)**	**P value**
**High total cholesterol**	23 (1.6)				
Unadjusted		3.9 (-0.5 to 8.4)	0.085	1.8 (-1.8 to 5.5)	0.320
Multivariate^b^		4.4 (-0.5 to 9.3)	0.080	1.8 (-2.2 to 5.8)	0.880
**High triglycerides**	147 (10.2)				
Unadjusted		4.3 (2.4 to 6.1)	<0.001	3.7 (2.2 to 5.2)	<0.0001
Multivariate^b^		4.1 (2.1 to 6.0)	<0.001	3.6 (2.1 to 5.2)	<0.0001
**Low HDL-cholesterol**	1046 (72.3)				
Unadjusted		-0.6 (-1.8 to 0.7)	0.374	-0.7(-1.6 to0.3)	0.190
Multivariate^b^		-0.5 (-1.8 to 0.8)	0.464	-0.5 (-1.6 to 0.6)	0.351
**Raised fasting glucose**	15 (1.3)				
Unadjusted		11.8 (6.2 to 17.4)	<0.001	8.0 (3.5 to12.6)	<0.0001
Multivariate^b^		10.0 (4.6 to 15.4)	<0.001	6.9 (2.5 to11.3)	<0.0001
**High HbA**_**1**_**c**	9 (0.6)				
Unadjusted		14.5 (7.4 to 21.6)	<0.001	9.5 (3.7 to 15.3)	0.003
Multivariate^b^		14.0 (6.9 to 21.0)	<0.001	9.3 (3.5 to 14.9)	0.004
**High body mass index**	214 (12.7)				
Unadjusted		6.9 (5.3 to 8.4)	<0.001	5.1 (3.9 to 6.3)	<0.0001
Multivariate^b^		6.7 (5.0 to 8.4)	<0.001	4.8 (3.5 to 6.2)	<0.0001
**High waist circumference**	234 (14.0)				
Unadjusted		6.6 (5.0 to 8.1)	<0.001	5.3 (4.1 to 6.5)	<0.0001
Multivariate^b^		6.2 (4.6 to 7.8)	<0.001	5.2 (3.9 to 6.4)	<0.0001

Missing for total cholesterol = 232, triglycerides =232, HDL cholesterol = 232, fasting blood glucose = 483, HbA_1_c = 234, BMI = 10, waist circumference = 2.

^a^Cut-offs for high total cholesterol = 5.17 mmol/L, high triglycerides =1.7 mmol/L, low HDL cholesterol =1.04 mmol/L, high BMI = 23, abdominal obesity = waist circumference ≥80 cm, Raised fasting glucose = 5.6 mmol/L and high HbA_1_c = 7. Those with normal values were considered as the reference group.

^b^Analysis done using simple and multivariate linear regression. The multivariate models adjusted for age, cigarette smoking, alcohol consumption, household farming and food assets, ethnicity, and occupation.

^c^Beta coefficients represent the mean difference in systolic or diastolic blood pressure comparing those with abnormal values of each predictor variable to those with normal values.

These differences in SBP and DBP were statistically significant (p ≤ 0.01) for TG, HbA_1_c, fasting glucose, BMI and WC. The greatest differences were between women with either high HbA_1_c (SBP β = 14.5 mm Hg; DBP β = 9.5 mm Hg) or raised fasting glucose (SBP β = 11.8 mm Hg; DBP β = 8.0 mm Hg) compared to women with normal values. Though the beta coefficients were slightly attenuated in multivariate models controlling for factors found to be associated with hypertension in the full cohort, the association with TG, HbA_1_c, fasting glucose, BMI and WC remained highly significant. TG, fasting glucose and HbA_1_c remained significantly associated with both SBP and DBP even after further adjustment for BMI (data not shown).

## Discussion

In this sample of relatively young, rural Nepalese women, the prevalence of hypertension and pre-hypertension was 3.3% and 14.4%, respectively. Household SES was associated with hypertension, notably indicators of household farming assets and food storage. Hypertension was more prevalent among older women, smokers and alcohol drinkers, but significantly less prevalent among women who worked outside the home. Although a low percentage of women had high total cholesterol, 73% of women had low HDL cholesterol and about 10% had high triglycerides. BP was not associated with cholesterol levels, but was strongly associated with triglycerides, BMI, WC, fasting glucose and HbA_1_c.

A number of reports suggest that cardiovascular disease and hypertension are rapidly increasing both in urban and rural areas of South Asia [3,4,18,19], yet there have been few population based studies and prevalence estimates vary widely. The only evidence from rural Nepal comes from a 1981 study in which the prevalence of hypertension among people aged 20 or more was about 6% in the hill areas and 8% in the plains [20]. The reported prevalence of hypertension in rural areas of India, Bangladesh and Pakistan is in the range of 4.5 - 22% [21-25]. These studies varied in the included age groups, study settings, and criteria for classifying hypertension. Nevertheless, the prevalence of hypertension among our cohort based on a cut-off of 140/90 mm Hg was 3.3%, one of the lowest reported estimates among rural communities in South Asia. One likely explanation is the young age of our participants, in contrast to many of the aforementioned studies which were among older participants. None of the previous studies reported the prevalence of pre-hypertension. Individuals with pre-hypertension have a high likelihood of progression to hypertension over the subsequent 5 years [26]. We have also seen a significant positive association between age and prevalent hypertension and it is likely that many of the women who currently have pre-hypertension will progress to hypertension as they age. Moreover, pre-hypertension itself is also a risk factor for cardiovascular disease and cardiovascular mortality [27].

To explore the determinants of hypertension, several researchers have investigated the association with SES. In rural India and Bangladesh, most studies reported a positive relationship between SES and hypertension [22,25,28,29], while only one found the opposite [30]. Defining SES is complex in the developing world. Many studies combined data on education, occupation, housing quality, land ownership, durable goods, income and livestock to create a composite index of SES. Creating an overall index in this manner might not reflect the true relationship between SES and hypertension consistently as different components in the index could have varying influences on hypertension [31]. To capture this variability we created SES indices on three different aspects. The SES component representing household farming assets and food storage (such as land, goats, cattle, bullock carts, and per capita amount of rice stored in the household) was negatively associated with hypertension. This SES component was also negatively associated with pre-hypertension for Madheshi women. This finding might suggest that in this rural setting in Nepal, those who are more food insecure are more vulnerable to non-communicable diseases, a finding that has been reported in developed countries [32,33]. The other two aspects of SES, household quality and household assets, were not associated with hypertension but showed a negative and significant relationship with pre-hypertension for Pahadi women. While it is unclear why two of the factors were more strongly associated within one group and the third factor more strongly associated within the other ethnic group, the general pattern appears to be that low SES is associated with high blood pressure for women of both the groups. Our findings do not support the finding from other studies in rural South Asia where higher SES were associated with increased prevalence of hypertension. Rather, these findings indicate that in the context of rural women in a low income country, having a higher SES is a protective factor for health. We examined literacy and occupation as separate variables from the SES scores in our multivariate models. We observed that those working outside home had a lower risk of hypertension than those who did not work or worked in the home. While it is unknown why this relationship may exist, we speculate that this variable might be a proxy for regular physical activity or it may suggest that women going outside of the home regularly have more chance to interact with different people and are more aware of health irrespective of their literacy or SES.

We have found that a total of 72.3% of women had low HDL cholesterol, a pattern of dyslipidemia that was also observed in studies mainly from India, where the prevalence of low HDL cholesterol has ranged from 41-68% [34-37]. These studies also reported that the prevalence of high TG was between 10-48%. In addition, a recent study of urban Nepalese women reported a similar pattern-- 40% of them had lower HDL cholesterol and 36% had high TG [38]. Such lipid patterns observed in South Asia are different from patterns in Western countries. By comparison, a nationally representative sample from the United States reported that 30% of females had low HDL cholesterol and 21% had high TG [39]. Some of the apparent difference between regions could be explained by the wide usage and availability of lipid lowering drugs in developed world or could be reflective of a distinct South Asian diet pattern largely dependent on carbohydrates, which may contribute to hypertriglyceridemia and lower HDL cholesterol [40].

The prevalence of raised fasting glucose was somewhat lower in our study (1.3%) than other reports from rural Nepal and India (~3.5%) [41-43]. We found that 14.0% women had abdominal obesity, lower than the 32% prevalence reported by another study of rural Indian women [44] and 50% prevalence of urban Nepali men [45]. Yet, most of the measured metabolic factors (TG, BMI, WC, fasting plasma glucose and HbA_1_c) were positively and strongly associated with blood pressure. The strongest relationship was for glucose abnormalities (fasting glucose and HbA_1_c) -- women with high glucose or high HbA_1_c had a mean SBP that was 11–14 mm Hg higher than those with normal values. These findings agree with previous investigations on the tendency of hypertension to occur concurrently with other metabolic disorders [46-48]. The combination of hypertension with lipid abnormalities, glucose intolerance and abdominal obesity comprise metabolic syndrome [49], which increases the risk of cardiovascular diseases and diabetes [50].

Our evidence suggests that modifiable risk factors like obesity, lipid abnormalities and glucose intolerance tend to coexist with raised BP and may begin in early life. This finding implies that screening for and managing multiple risk factors should be considered instead of only a single one. In contexts such as these, early life malnutrition may also be associated with an elevated risk of CVD, particularly if followed by later life overweight or obesity [51]. It is important, therefore, to consider health and nutrition risk factors across the life course.

## Conclusion

Although the prevalence of hypertension was low in this relatively young cohort, nearly 18% were classified as either pre-hypertensive or hypertensive. We found a negative association between SES and hypertension, which could be an indication of the maturation of CVD epidemic in that region. This epidemic has to be prevented because across the South Asian region, it has been predicted that if rigorous and early preventive measures are not taken, treatment costs of CVD will become a substantial burden on national economies [19]. It is also predicted that a reduction in the population distribution of systolic blood pressure of 2 mm Hg results in 6%, 4% and 3% reductions in 1-year stroke related mortality, coronary heart disease related mortality and overall mortality, respectively [52]. It also has been projected that reducing chronic disease mortality even by only 2% per year by 2015 could save 10% of the expected loss of income and around $8 billion collectively in South Asian and other middle or low income countries [19]. We have shown that different cardiovascular risk factors including blood pressure tend to be strongly associated with each other. Identification of these risk factors at an early stage of life is an important opportunity for primary prevention of hypertension through lifestyle modification to prevent disease progression. We hope that these data will contribute to the evidence of CVD risk in a poor, rural population, helping to devise policies to prevent disease in this rural population of Nepal as well as in other similar settings in South Asia.

## Competing interests

The authors’ declare that they have no competing interest.

## Authors’ contributions

RJK, CPS, PC and KPW conceptualized the analysis plan for this paper. RJK and CPS wrote the paper and performed the statistical analysis together with LSFW. CPS, SCL and SKK were involved in the data collection and project management. KJS oversaw the laboratory analysis and reviewed the manuscript. KPW served as the PI for the original study and subsequent follow-up. All authors have reviewed and approved the final manuscript.

## Pre-publication history

The pre-publication history for this paper can be accessed here:

http://www.biomedcentral.com/1471-2458/13/55/prepub
